# The association between pre pregnancy body mass index and risk of preeclampsia: a registry based study from Tanzania

**DOI:** 10.1186/s12884-018-1687-3

**Published:** 2018-02-21

**Authors:** Dorah Mrema, Rolv Terje Lie, Truls Østbye, Michael Johnson Mahande, Anne Kjersti Daltveit

**Affiliations:** 1 Department of Global Public Health and Primary Care, Faculty of Medicine, University of Bergen, Postboks 7804, N-5020 Bergen, Norway; 2 Department of Obstetrics and Gynaecology, Kilimanjaro Christian Medical Centre, Moshi, Tanzania; 3 Norwegian Institute of Public Health, Bergen, Norway; 4 Institute of Public Health, Department of Epidemiology & Biostatistics, Kilimanjaro Christian Medical University College, Moshi, Tanzania

**Keywords:** Preeclampsia, Obesity, BMI, Low income countries, Birth registry, Tanzania

## Abstract

**Background:**

Preeclampsia is among the leading causes of maternal mortality and morbidity worldwide, occurs in 2-8% of all pregnancies, and is estimated to account for at least 9 % of maternal deaths in Africa. Studies from developed countries show that high pre pregnancy body mass index (BMI) increases the risk of preeclampsia. We examined the association between pre pregnancy BMI and the risk of preeclampsia in Tanzania, a low income country.

**Methods:**

Data from the Kilimanjaro Christian Medical Center (KCMC) Medical Birth Registry recorded between July 2000 and May 2013 were used. We restricted the study population to singleton deliveries among women with no or one previous pregnancy. Pre pregnancy BMI (kg/m^2)^ was categorized according to the WHO categories of underweight (less than 18.5), normal (18.5 – 24.9), overweight (25.0 – 29.9) and obese (30 or more). Potential confounders were adjusted for in multivariable analyses.

**Results:**

Among the 17,738 singleton births, 6.6% of the mothers were underweight, 62.1% were of normal BMI, 24.0% were overweight, and 7.3% were obese. Five hundred and eighty-two pregnancies (3.3%) were affected by preeclampsia. Compared to those with normal BMI, overweight and obese women had a higher risk of preeclampsia (aOR (95% CI) 1.4 (1.2 – 1.8) and 1.8 (1.3 – 2.4)), respectively, while underweight women had a lower risk (0.7 (0.4-1.1)).

**Conclusions:**

Pre pregnancy maternal overweight and obesity were associated with an increased risk of preeclampsia in Tanzania. Risks were similar to those reported in high income countries.

## Background

Preeclampsia is a serious complication affecting 2-8% of all pregnancies. Globally, more than 287,000 women die each year due to pregnancy related causes [1], of which 10-15% are estimated to be due to preeclampsia. Most maternal deaths occur in developing countries. Millennium Development Goal number five is to reduce maternal mortality by three quarters by 2015. Given the high number of maternal deaths in low income countries due to preeclampsia, both prevention of preeclampsia and optimal management of preeclamptic pregnancies are important to further reduce maternal mortality [2].

The etiology of preeclampsia remains unclear, but mechanisms related to the placenta, genes, immune response, insulin resistance, and maternal vascular disease are suggested to contribute [3–6]. Established risk factors for preeclampsia include nulliparity, advanced maternal age, overweight/obesity, chronic hypertension, diabetes, previous preeclampsia, family history of preeclampsia, long time since previous pregnancy, and multiple pregnancy [7]. Obesity has been associated with a 2-4 fold increased risk of preeclampsia in different populations [8–12], and is a leading identified attributable risk for this disorder. A population based study from Dar Es Salaam, Tanzania, reported that the prevalence of obesity among women of reproductive age increased progressively from 3.6% in 1995 to 9.1% in 2004 [13]. The Tanzanian Demographic Health Survey for the years 2004 and 2005 reported a prevalence of 13% and 4%, respectively of overweight and obesity among women of reproductive age [14].

Since clinical records of births are lacking or not suitable for research in many African countries, studies on preeclampsia in women of African descent have been mostly based on immigrants to high income countries or descendants of immigrants [15]. Some studies have reported that women of African origin are at increased risk of preeclampsia, but it is not clear to which extent this is explained by the presence of specific risk factors for preeclampsia. Also studies on the association between preeclampsia and obesity are mainly based on women in resource rich countries [16].

Etiology, epidemiology and cultural significance of overweight and obesity likely vary from population to population. There are also indications that the course and outcome of preeclamptic pregnancies differs by race and ethnicity [17]. These aspects call for collection of high quality data to study overweight and obesity as risk factors of preeclampsia in indigenous African women. We aimed to examine the association between pre pregnancy BMI and development of preeclampsia in a low income setting in Tanzania. A secondary aim was to explore to which extent this association can be explained by maternal disease before pregnancy (hypertension, heart disease, diabetes).

## Methods

### Study design, setting, source of data and population

**Fig. 1:**
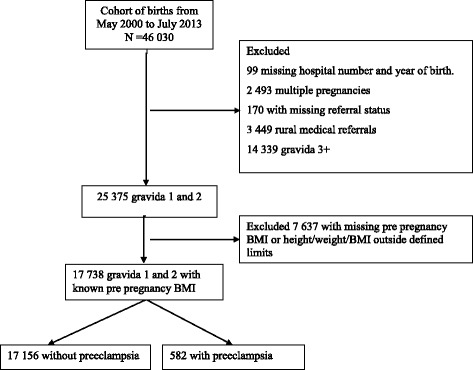
Schematic presentation of the study cohort. Data from the Kilimanjaro Christian Medical Centre (KCMC) birth registry

This is a registry-based study using existing prospectively collected birth registry data from Kilimanjaro Christian Medical Centre (KCMC). KCMC is a zonal hospital based in Moshi urban district, Kilimanjaro region in Northern Tanzania. The medical birth registry at KCMC was established in collaboration with researchers at the Department of Global Public Health and Primary Care (formerly Department of Public Health and Primary Health Care) at the University of Bergen Norway, and has been in operation since July 2000. The data were collected by obstetricians and midwives from all women who delivered at KCMC from July 2000 to May 2013. Trained nurse midwives conduct a face to face interview in the hospital, using a standardized questionnaire for all mothers within 24 h after delivery, or later in case of caesarean section or other complications [18]. The questionnaire is based on check boxes and text boxes. Since the mothers are discharged within 24 h after a normal delivery, the interviews are done on a daily basis including public holidays and weekends. Abstracted data from files relating to each mother, written by obstetricians, are also included. In addition, mothers admitted to the hospital are asked to provide their antenatal (ANC) cards for further clarification regarding their pregnancy records including pre pregnancy weight and height. This should limit the possibility of recall bias. In summary, the information collected during interview and through inspection of medical files included parents’ social-demographic characteristics, reproductive history, pregnancy and birth characteristics such as; maternal health before pregnancy, maternal health during pregnancy, and complications during labour and delivery, and newborn health status. Our sample includes mothers who delivered at Kilimanjaro Christian Medical Centre (KCMC) from July 2000 to May 2013. The mother’s records were linked to their children’s records using a unique maternal hospital number assigned for each woman who deliver at KCMC for the first time. A total of 46,030 deliveries were recorded. We restricted the study population to women with no or one previous pregnancy. Definition of pregnancy order was based on information about previous pregnancies in the questionnaire including pregnancies lasting less than 28 weeks. Compared to a selection based on previous stillbirths and live births only, exclusion of such early losses reduced the study population by 6 %. Exclusion of women with more than one previous pregnancy was done in order to focus on baseline BMI and not BMI as a result of high parity. We excluded multifetal pregnancies, women who were referred for delivery at KCMC from the rural area for medical reasons, and those with missing information on weight or height (Fig. 1). Our final sample consisted of 17,738 singleton births.

### Study variables and definitions

The main outcome of interest was preeclampsia. In the registry’s manual, preeclampsia is defined as gestational hypertension of at least 140/90 mmHg, measured on two separate occasions at least four hours apart, and accompanied by proteinuria, arising after the 20th week of gestation in a previously normotensive woman. This includes mild preeclampsia (blood pressure lower than 160/110) and severe preeclampsia (blood pressure 160/110 or higher). The main exposure variable was pre pregnancy BMI based on maternal pre pregnancy weight in kilograms and maternal height in centimetres from antenatal care (ANC) visits. If weight was lacking in the record, self-reported weight was used if reasonable. In a secondary analysis we used gestational age at delivery as a proxy for preeclampsia severity and analysed preeclampsia in connection with term delivery (gestational age 37 or more weeks) and preeclampsia in connection with preterm delivery (gestational age below 37 weeks), as separate outcomes. We excluded women whose records had a height < 130 cm or > 200 cm and women whose records had a weight < 35 kg or > 120 kg. BMI was calculated as body weight in kg/height in metres squared, and we excluded records with BMI above 40 (0.4%) and BMI below 15 (0.5%). Exclusions of records based on recorded height, weight and BMI were performed to reduce potential effects of typing errors. We categorised BMI according to WHO definitions as underweight <18.5, normal weight 18.5-24.9, overweight 25.0-29.9, and obese ≥30.

### Data analysis

Descriptive statistics including means and proportions were calculated. Pearson Chi-square statistics was used to assess associations between BMI categories and categorical factors, and between categorical factors and preeclampsia.. The significance level was set at *p* ≤ 0.05 (2-tailed). Binary and multivariable logistic regression analysis was performed to assess the strength of association between the independent variables and preeclampsia. Unadjusted and adjusted odds ratios with 95% confidence intervals were reported. The ordinal variables (mother’s BMI, age, education and height) were also included as continuous covariates in the model to explore a possible dose-response relationship with preeclampsia. *P*-values for trend were reported from this analysis.

We a priori selected a set of potential confounders (mother’s age, number of previous pregnancies, mother’s education, antenatal care visits, marital status, mother’s occupation, mother’s tribe, and mother’s height). In a second step we included some accompanying medical conditions in the multivariable model; chronic hypertension, diabetes mellitus, and heart diseases before pregnancy. These factors may be a result of the main exposure BMI and should not be considered as pure confounders. However, since they are known to be associated with both BMI and preeclampsia, we wanted to explore the extent to which the association between BMI and preeclampsia was explained by these factors.

All the selected potential confounders were associated with preeclampsia with a *p*-value less than 0.1 in the univariate analysis and were included in the final model. Records with missing values were included only in the descriptive analysis of the participant characteristics. Since for each variable in the multivariable model the proportion of missing values was less than 1%, individuals with missing values on any independent variable were not included in the model. We also tested for interaction between maternal height (< 160 cm vs ≥ 160 cm) and BMI, and between gestational age (< 37 weeks, ≥ 37 weeks) and BMI, in their impact on risk of preeclampsia, by adding an interaction term in the model. In the interaction analyses BMI was included as a continuous variable.

We also used a clustered analysis technique with robust estimation of variances to account for correlation between successive births from the same mother.

Data were analysed using Statistical Package for Social Sciences (SPSS) version 20 for Windows.

## Results

Of the 17,738 singleton births, overall pre pregnancy mean BMI was 23.5, 6.6% were births to underweight mothers, 62.1% to normal weight mothers, 24.0% to overweight mothers and 7.3% to obese mothers (Table 1). The highest mean BMI and highest proportion with obesity were found among women above 35 years of age, women who had the highest education, women with missing information on marital status, business or professional women, women from the Chagga tribe, and women with four or more ANC visits. Differences in mean BMI were modest, and only among mothers above 35 years of age was mean BMI above 25. The highest proportion with underweight was found among teenage mothers, mothers with missing information on education, women without partner, students, mothers from tribes other than Chagga and Pare, mothers who were missing information on ANC visits, and mothers with a height ≥ 165 cm. Women having their second child and women with chronic hypertension or gestational hypertension or diabetes before pregnancy had a higher mean BMI and a higher proportion of overweight and obesity than women having their first child and women without these conditions (Table 2).

**Table 1:** Sociodemographic characteristics of the participants by BMI (kg/m^2^) category; 17,738 singleton first or second deliveries. KCMC Medical birth registry July 2000-May 2013

Characteristics	N	Mean (SD) BMI	BMI < 18.5 Underweight	BMI 18.5-24.9 Normal	BMI 25.0-29.9 Overweight	BMI >= 30 Obese	χ2 *p*-value
Overall (n)	17,738		1178	11,008	4258	1294	
%		23.5	6.6	62.1	24.0	7.3	
Mothers age (yrs)							< 0.001
13-19 20-24 25-29 30-34 35-50 Missing	2151 6437 5755 2606 755 34	21.8 (3.2) 22.8 (3.6) 24.0 (4.0) 24.9 (4.2) 25.6 (4.4) 23.9 (3.6)	12.6 7.9 5.1 3.2 2.9 -	72.1 68.3 58.3 51.0 46.5 64.7	13.5 19.5 27.7 33.0 33.8 23.5	1.8 4.3 8.9 12.9 16.8 8.8	
Mothers education							< 0.001
None Primary Secondary (8-11 yrs) Higher (12+ yrs) Missing	177 9116 1143 7277 26	22.8 (3.3) 23.2 (3.8) 23.2 (4.0) 24.0 (4.1) 23.5 (3.4)	9.0 7.2 8.7 5.6 11.5	69.5 75.5 62.6 57.6 73.1	18.1 21.4 23.1 27.6 15.4	3.4 6.0 6.7 9.1 -	
Marital status							< 0.001
With partner Without partner Missing	14,807 2866 65	23.6 (3.9) 23.1 (4.1) 24.3 (4.4)	6.2 8.9 4.6	61.8 63.4 58.5	24.7 20.7 24.6	7.3 7.0 12.4	
Mother’s occupation							< 0.001
Housewife Farmer Service Business Professional Student Missing	3567 2784 1286 4125 3790 592 1594	23.0 (3.7) 22.5 (3.5) 23.8 (3.9) 24.2 (4.2) 24.2 (4.0) 22.4 (3.8) 23.1 (3.8)	8.2 9.1 5.2 5.4 4.4 12.0 6.8	65.9 70.0 59.9 56.7 57.0 65.4 66.1	20.8 17.3 27.4 27.3 29.1 17.9 21.9	5.1 3.6 7.5 10.7 9.6 4.7 5.2	
Mothers tribe							< 0.001
Chagga Pare Other	10,164 2044 5530	23.8 (4.0) 23.5 (4.0) 23.0 (3.9)	5.4 7.3 8.7	60.2 62.2 65.4	26.1 23.6 20.2	8.2 6.8 5.7	
Antenatal care							< 0.001
< 4 ≥ 4 Missing	8742 8783 213	23.4 (4.0) 23.7 (3.9) 23.1 (3.7)	7.3 5.9 10.3	63.4 60.7 60.1	22.3 25.7 24.9	7.0 7.6 4.7	
Mother’s height							< 0.001
< 155 cm 155 cm-164 ≥ 165 cm	4142 9760 3836	23.8 (3.9) 23.5 (4.0) 23.0 (4.0)	5.3 6.2 9.1	64.5 62.6 58.0	23.6 23.5 25.9	6.6 7.7 7.0

**Table 2:** Maternal health related characteristics in by BMI (kg/m^2^) category; 17,738 singleton first or second deliveries. KCMC Medical birth registry July 2000-May 2013

Maternal health characteristics	N	Mean (SD) BMI	BMI < 18.5 Underweight	BMI 18.5-24.9 Normal	BMI 25.0-29.9 Overweight	BMI >= 30 Obese	χ2 *p*-value
Overall (n)	17,738		1178	11,008	4258	1294	
%		23.5	6.6	62.1	24.0	7.3	
Pregnancy order							< 0.001
First Second	10,354 7384	22.9 (3.7) 24.3 (4.1)	8.2 4.5	66.4 56.0	20.5 29.0	5.0 10.5	
Chronic hypertension							< 0.001
Yes No	76 17,662	25.5 (4.9) 23.5 (3.9)	5.3 6.6	40.8 62.2	35.5 24.0	18.4 7.2	
Gestational hypertension							0.02
Yes No	41 17,697	25.0 (4.1) 23.5 (4.0)	- 6.7	48.8 62.1	41.5 24.0	9.8 7.3	
Diabetes*							0.13
Yes No	21 17,717	24.9 (4.6) 23.5 (4.0)	9.5 6.6	42.9 62.1	28.6 24.0	19.0 7.3	
Heart disease*							0.10
Yes No	53 17,685	22.5 (4.5) 23.5 (4.0)	15.1 6.6	54.7 62.1	22.6 24.0	7.5 7.3	
Gestational age							< 0.001
Below 37 weeks 37 or more weeks Missing	2630 13,675 1433	23.1 (4.0) 23.6 (3.9) 23.4 (4.0)	8.5 6.2 6.7	64.0 61.7 61.5	20.8 24.7 23.2	6.7 7.4 7.5	

*Before pregnancy

Preeclampsia was recorded for five hundred and eighty-two pregnancies (3.3%)**.** The risk of preeclampsia increased with increasing pre pregnancy BMI, maternal age, maternal educational level, and body height (*p*-values for trend < 0.05) (Table 3). Preeclampsia was more common among married women, among those working in service or professional workers, and among women from the Pare tribe.

**Table 3:** Observed and adjusted odds ratio of preeclampsia according to risk factors;17,738 singleton first or second deliveries. KCMC Medical birth registry July 2000-May 2013

Maternal characteristics	No preeclampsia (n)	Preeclampsia [n (%)]	Crude OR (95% CI) *P*-value		Adjusted* OR (95% CI) *P*-value	
Mother’s BMI (kg/m^2^)				*< 0.001*		*< 0.001*
< 18.5 underweight	1153	25 (2.1)	0.7 (0.5-1.1)		0.7 (0.4-1.1)	
18.5-24.9 normal	10,694	314 (2.9)	Ref		Ref	
25.0-29.9 overweight	4077	181 (4.3)	1.5 (1.3-1.8)		1.4 (1.2-1.8)	
30 thru highest obese	1232	62 (4.8)	1.7 (1.3-2.3)		1.8 (1.3-2.4)	
*P for trend*			*< 0.001*		*< 0.001*	
Mother’s age				*< 0.001*		*< 0.001*
13-19	2090	61 (2.8)	1.1 (0.8-1.5)		0.9 (0.7-1.3)	
20-24	6268	169 (2.6)	Ref		Ref	
25-29	5568	187 (3.2)	1.2 (1.0-1.5)		1.2 (1.0-1.5)	
30-34	2493	114 (4.4)	1.7 (1.3-2.2)		1.6 (1.2-2.1)	
35-50	706	49 (6.5)	2.6 (1.9-3.6)		2.6 (1.8-3.7)	
*P for trend*			*< 0.001*		*< 0.001*	
Mother’s education				*0.08*		*0.4*
None	172	5 (2.8)	0.8 (0.3-1.9)		1.2 (0.5-3.1)	
Primary	8839	277 (3.0)	0.8 (0.7-1.0)		1.1 (0.9-1.5)	
Secondary (8-11)	1111	31 (2.7)	0.7 (0.5-1.1)		0.9 (0.6-1.3)	
Higher (12+)	7009	268 (3.7)	Ref		Ref	
*P for trend*			*0.02*		*0.2*	
Pregnancy number				*0.06*		*0.001*
First	9992	362 (3.5)	Ref		Ref	
Second	7164	220 (3.0)	0.8 (0.7-1.0)		0.7 (0.6-0.9)	
Marital status				*0.003*		*0.02*
With partner	14,348	459 (3.1)	Ref		Ref	
Without partner	2746	120 (4.2)	0.7 (0.6-0.9)		0.8 (0.6-1.3)	
Mother’s occupation				*< 0.001*		*0.07*
Housewife	3463	104 (2.9)	1.0 (0.8-1.4)		1.0 (0.8-1.4)	
Farmer	2704	80 (2.9)	Ref		Ref	
Service	1227	59 (4.6)	1.6 (1.2-2.3)		1.4 (1.0-2.0)	
Business	4012	113 (2.7)	1.0 (0.7-1.3)		0.8 (0.6-1.1)	
Professional	3629	161 (4.2)	1.5 (1.1-2.0)		1.2 (0.9-1.7)	
Student	573	19 (3.2)	1.1 (0.7-1.9)		1.1 (0.6-1.8)	
Mother’s tribe				*< 0.001*		*< 0.001*
Chagga	9874	290 (2.9)	Ref		Ref	
Pare	1951	93 (4.5)	1.6 (1.3-2.1)		1.9 (1.4-2.4)	
Others	5531	199 (3.6)	1.3 (1.1-1.5)		1.5 (1.2-1.8)	
Mother’s height				*0.009*		*0.01*
< 155 cm	4017	125 (3.0)	0.7 (0.6-0.9)		0.7 (0.6-1.0)	
155-164 cm	9459	301 (3.1)	0.8 (0.6-0.9)		0.7 (0.6-0.9)	
≥ 165 cm	3680	156 (4.1)	Ref		Ref	
*P for trend*			*0.01*		*0.02*	

*All variables are in the multivariable model

After adjustment**,** overweight and obese women were 1.4 and 1.8 times more likely to have preeclampsia than women with normal BMI (95% CI 1.2-1.8 and 1.3-2.4, respectively), while underweight women were less likely to have preeclampsia (adjusted OR 0.7, 95% CI 0.4-1.1). Mother’s age, marital status and tribe remained associated with preeclampsia after adjustment, and *p*-values for trend were significant for pre pregnancy BMI, mother’s age, and mother’s height.

We further adjusted for selected medical conditions known to be associated with both BMI and preeclampsia, i.e. chronic hypertension, heart disease and diabetes before pregnancy. The effect of BMI then changed slightly from 1.8 in the highest BMI class to 1.7 (data not presented in table).

The association between BMI and preeclampsia was slightly stronger among mothers delivering at term than among mothers delivering preterm; adjusted OR (CI) per unit increase in BMI 1.08 (1.05-1.11) vs 1.05 (1.01-1.08), *p*-value for interaction 0.15.

The association between BMI and preeclampsia was slightly stronger among mothers with a height below 160 cm (45% of the women) compared to mothers with a height 160 cm or above; adjusted OR (CI) per unit increase in BMI 1.08 (1.04-1.11) vs. 1.05 (1.03-1.09), *p*-value for interaction 0.40.

A total of 1499 women were recorded with two pregnancies in the study population. Results from a clustered analysis accounting for correlation between births from the same mother were almost identical to the presented results (data not shown).

## Discussion

We found a positive association between increasing pre pregnancy body mass index and the risk of developing preeclampsia, amounting to an adjusted odds ratio of 1.8 for obese women with BMI above 30 as compared to normal weight women with BMI between 20 and 24.9. Among the maternal characteristics included in our analysis, only maternal age above 35 years of age showed a higher odds ratio. Our findings are in line with previous studies based on populations of pregnant women in high income countries [8–12].

Using the WHO definition of overweight and obesity, the prevalence of pre pregnancy overweight and obesity in our study population of ethnic African women was 24.0% and 7.3%, respectively. This compares with a study from Dar Es Salaam, where prevalence of obesity among females of reproductive age increased from 3.6% in 1995 to 9.1% in 2004 [13]. Our results, with nearly one third of the women were overweight or obese, correspond with global numbers of obesity, showing that obesity has now become a significant health challenge also in many low income countries [19].

We had no information on severity of preeclampsia or time of onset, but used preterm birth as a proxy for severity. The association between increasing BMI and preeclampsia was strongest for preeclampsia in connection with a term delivery, although the interaction between gestational age at delivery and preeclampsia was not statistically significant. These results are consistent with a study from the Swedish birth registry where the association between increasing BMI was stronger for term preeclampsia than for preeclampsia before term [20]. A possible explanation for these findings is that early and severe preeclampsia more often originates in placenta, whereas late and mild preeclampsia is more related to metabolic disease and hence more often associated with high BMI [21].

In our data, being overweight and obese was associated with higher maternal age, being married, high education, and being from the Chagga tribe, the majority tribe in the area. This indicates that overweight and obesity in this population are associated with higher socioeconomic status rather than low socioeconomic status which is the case in resource rich countries. In our study, adjustment for socioeconomic factors had, however, little influence on the effect of BMI. Socioeconomic factors are not among major risk factors of preeclampsia [7], and, although associated with BMI, are therefore not likely important confounders.

Our study was based on women of African origin in a low income setting, but we found an association between BMI and preeclampsia that was similar both in direction and magnitude to those from resource rich countries. However, the course and outcome of a preeclamptic pregnancy may vary not only by race or ethnicity but also by available resources. It is therefore important that African women, who bear a disproportionate burden of global maternal morbidity and mortality due to preeclampsia and other pregnancy complications, are included in studies on preeclampsia.

### Strengths and limitations

A strength of our study is that we used data from a registry with a systematic collection of data based on a structured interview during the 13 years study period. It is also a strength that we had information on several possible confounding factors such as socio-demography and maternal disease both before and during pregnancy.

Because the study is hospital based we cannot rule out selection bias if women who deliver at KCMC differ from women in the area who deliver at home or in other hospitals. In the Kilimanjaro region, 13% of all deliveries take place outside a health facility, and nearly all women receive antenatal care from a skilled provider [22]. In general, selection will mostly influence prevalence estimates of exposure and outcome and to a lesser extent effect estimates. As a result of possible selection to giving birth at KCMC and also of how we selected our study population (exclusion of multifetal deliveries and women from rural areas who were referred to KCMC for medical reasons), the preeclampsia rate of 3.3% may not reflect the rate in the population. Furthermore, poor ascertainment of the mildest forms of preeclampsia may influence the observed preeclampsia rate. Among women in Northern Tanzania who had attended ANC for their most recent birth in the last five years, 79.9% had their blood pressure measured and 65.4% had their urine tested [22].

The mother’s weight was retrieved from her antenatal record if her first antenatal visit took place before week 16 of pregnancy, otherwise self-reported weight was recorded if reasonable. Most studies report that women tend to underreport their body weight [23], but this might vary from population to population depending on how socially acceptable or desirable it is to be underweight or overweight. However, since body weight was reported before the onset of preeclampsia, reporting error in any direction most likely represents a non-differential misclassification and therefore will tend to change the odds ratios towards 1, i.e. give conservative effect estimates. Furthermore, unmeasured factors such as nutrition and diet might represent residual confounding and affect our results if associated with both body mass index and preeclampsia.

Our main aim was to assess the association between BMI and preeclampsia, but we also report associations between the covariates and preeclampsia. We acknowledge that multiple comparisons are a concern and that the additional tests should be regarded as exploratory.

## Conclusions

‘There appears to be an association between increased pre pregnancy body mass index category and increased preeclampsia risk, in this resource limited population. The increasing prevalence of obesity in pre pregnant women in low income countries hinders efforts to improve perinatal health and reduce maternal mortality. Close clinical antenatal monitoring of all pregnant women in Tanzania, especially blood pressure monitoring, is critically important, but especially so for overweight and obese women.

## Abbreviations

aOR: adjusted odds ratio
BMI: body mass index
OR: odds ratio
WHO: World Health Organization
